# Monitoring patient progress and changes in patient and clinician reported symptoms with measurement informed care in a pediatric primary care mental health integration program

**DOI:** 10.3389/fpsyg.2026.1898860

**Published:** 2026-10-14

**Authors:** Devin P. Adams, Jason Schweitzer, Jasmine R. Holt, Anne F. Bird, Nicole Carr-Lee, Kathryn A. Hollenbach

**Affiliations:** 1 Transforming Mental Health, Rady Children’s Hospital - San Diego, San Diego, CA, United States; 2 Department of Psychiatry, Child and Adolescent Division, University of California San Diego, San Diego, CA, United States; 3 Department of Pediatrics, University of California San Diego, San Diego, CA, United States

**Keywords:** measurement informed care, mental health, pediatrics, primary care integration, screeners

## Abstract

**Introduction:**

Measurement informed care (MIC) utilizes validated instruments to screen and monitor patient symptoms and has been shown to improve patient outcomes and streamline diagnostic processes. Our primary care mental health integration (PCMHI) program embedded mental health clinicians in the pediatric primary care setting to increase access to mental healthcare and provide care for adolescents. The purpose of this study was to identify changes in patients’ self-reported symptom severity and determine whether provider assessments of patient progress were associated with patients’ completed screening scores between the initial and final visits of short-term treatment within a PCMHI program.

**Methods:**

Patients were retrospective from a multisite pediatric PCMHI program. The program’s standardized measures included the Patient Health Questionnaire-9 (PHQ-9) and Generalized Anxiety Disorder-7 (GAD-7) for patients older than 12 years and the Pediatric Symptom Checklist-17 (PSC-17) for patients younger than 12 years. Before each PCMHI session, patients completed self-reported assessments, and after the session, clinicians provided a score reflecting overall patient progress. Data from treated patients were extracted from electronic medical records, and changes in patient scores were calculated and analyzed using Stata 16.1 (College Station, TX).

**Results:**

A total of 2,702 patients were eligible for analysis. Significant reductions (95% CIs) in the proportion of patients scoring above clinically relevant cutoffs were observed for the PHQ-9 (28.8%; 26.1–31.5%), GAD-7 (25.8%; 23.4–28.4%), PSC-17 attention subscale (16.5%; 14.6–18.6%), and PSC-17 internalizing subscale (17.3%; 15.3–19.4%) (all *p* < 0.001). No significant change was identified for the PSC-17 externalizing subscale (11.0%; 9.4–12.8%; *p* = 0.19). Effect sizes for score change among patients above the clinical cutoff were large (*r* = 0.68–0.80 across the PHQ-9 and GAD-7 severity scores), and the odds of moving below the cutoff exceeded the odds of moving above it for every measure except the externalizing subscale. CGI improvement scores were significantly correlated with the PHQ-9, GAD-7, and each of the PSC-17 subscales (all *p* < 0.001).

**Conclusion:**

Validated mental health assessments, completed by patients before their appointments, documented reductions in symptom severity over brief, short-term treatment and changes in patient- and clinician-completed assessments were significantly correlated. These results demonstrate the feasibility of using patient-provided self-assessments to monitor patient progress.

## 1 Introduction

Measurement informed care (MIC) uses validated assessments to screen for and monitor patient symptoms, identify patient needs, and enhance clinical care (Boswell et al., 2023; Lewis et al., 2019). Consistent measurement and clinician feedback have been shown to improve outcomes in youth mental health treatment (Bickman et al., 2011). Utilizing patient-reported measures at the beginning of the treatment and repeating them throughout care has been associated with improved patient engagement (Bickman et al., 2011; Jensen-Doss and Weisz, 2008; Scott and Lewis, 2015). MIC has also been associated with streamlined diagnostic processes and significant improvements in patient outcomes (Scott and Lewis, 2015).

Standardized screening tools and rating scales in mental health have gained traction over the last decade, providing objective insights into patients’ experiences, guiding treatment plans, and refining patient services (Jensen-Doss et al., 2020). Agreement between clinicians and patients or their parents/guardians is an important factor affecting attrition from treatment (Klein et al., 2010). These tools also help clinicians efficiently gather data that inform treatment decisions and track patients’ progress (Jensen-Doss et al., 2020).

Primary care mental health integration (PCMHI) models aim to provide behavioral and mental healthcare alongside physical care management in one setting. Integrated care in a primary care setting that addresses pediatric behavioral health needs has been shown to increase access to behavioral health treatment (Asarnow et al., 2015), and MIC monitoring has demonstrated behavioral and mental health changes associated with successful treatment. Few studies, however, have examined the association between patient-completed self-assessments and clinician assessments of change during the same encounter. We sought to examine these relationships in our PCMHI program.

A detailed description of the PCMHI program in this study can be found elsewhere (Schweitzer et al., 2023). In brief, PCMHI uses principles of Primary Care Behavioral Health (PCBH) (Reiter et al., 2018), with integrated health therapists (IHTs) embedded in affiliated pediatric primary care clinics to provide brief psychotherapeutic intervention. Patients with more complex needs are referred to child psychiatrists, who provide care at regional hub offices within the PCMHI program. These services may include consultation, assessment and short-term treatment, and medication management (Schweitzer et al., 2023). IHTs are typically master’s-level clinicians who provide evidence-based therapy in the primary care (4–6 visits) or hub (10–12 visits) settings (Schweitzer et al., 2023). Treatment is initiated by patients’ primary care providers, who refer patients to an IHT after identifying a mental health need (Schweitzer et al., 2023).

The purpose of this study was to determine whether screening score results reflected changes in symptom presentation and whether changes in patients’ self-reported assessments were associated with therapists’ assessment of patient progress.

## 2 Methods

We conducted a retrospective cohort study of patients referred for mental or behavioral health services to our PCMHI program between June 2020 and July 2024. This study was reviewed by The University of California San Diego Office of Institutional Review Board Administration (#807793) and was determined not to constitute human subject research. Changes in patients’ assessment scores between the initial and final visits were calculated for each of the assessments. Changes in actual scores and score categories were used for analyses.

Patients seen within the PCMHI program were treated by integrated health therapists embedded in their primary care clinics within the largest pediatric health network in San Diego and Riverside Counties. The clinics serve a mix of urban and suburban communities and a predominantly publicly insured population. Clinicians used evidence-informed treatments to address patients’ emotional and behavioral concerns. Common concerns, including anxiety, depression, and sleep problems, were frequently addressed using cognitive-behavioral therapy. Parent management training techniques were often used to address concerns regarding attention-deficit/hyperactivity disorder (ADHD), and parent–child interaction therapy was used to address externalizing or disruptive behaviors. In addition, clinicians used the First Approach Skills Training (FAST) program to treat symptoms of anxiety, depression, and traumatic experiences.

Before each visit, PCMHI patients completed validated self-reported measures covering symptom domains common in the PCMHI patient population. These included the Patient Health Questionnaire-9 (Kroenke et al., 2001) (PHQ-9), Generalized Anxiety Disorder-7 (Mossman et al., 2017; Spitzer et al., 2006) (GAD-7), and Pediatric Symptom Checklist-17 (Jellinek et al., 1986; Jellinek et al., 1999) (PSC-17). Clinicians also completed the Clinical Global Impression (Busner and Targum, 2007) (CGI) scale after each visit. The results of these measures were used to help guide therapeutic interventions, inform treatment planning, and support clinical decision-making. The PHQ-9 was used to measure and track depressive symptoms in patients aged 12 years and older (Liu and Adrian, 2019). Scores range from 0 to 27, with severity cutoffs defined as 0–4 for minimal, 5–9 for mild, 10–14 for moderate, 15–19 for moderately severe, and 20 or higher for severe depression (Kroenke et al., 2001). The GAD-7 measures general anxiety symptoms in patients aged 12 years and older, and scores range from 0 to 21 (Spitzer et al., 2006). Severity cutoffs are defined as 0–4 for minimal anxiety, 5–9 for mild anxiety, 10–14 for moderate anxiety, and 15 or higher for severe anxiety (Spitzer et al., 2006).

A score of >10 was used as the cutoff for dichotomous analyses of the PHQ-9 and GAD-7, with a score above 10 indicating an increased likelihood of major depressive disorder or generalized anxiety disorder, respectively (Kroenke et al., 2001; Spitzer et al., 2006). At this cutoff, the PHQ-9 has an overall sensitivity of 88% and specificity of 88%, whereas the GAD-7 has a sensitivity of 89% and specificity of 82%, respectively (Kroenke et al., 2001; Spitzer et al., 2006). The PSC-17 was used to assess behavioral health concerns in children younger than 12 years. It comprises three subscales: attention, internalizing, and externalizing (Gardner et al., 2007; Murphy et al., 2016). The attention and externalizing subscales use scores >7 to indicate elevated scores, whereas the internalizing subscale uses a cutoff of >5 (Murphy et al., 2016). The sensitivity of the PSC-17 ranges from 31 to 73%, and its specificity ranges from 74 to 96% depending on the disorder (Gardner et al., 2007).

The CGI is a clinician-rated measure of patient severity (CGI-S) and improvement (CGI-I) used by providers (Busner and Targum, 2007; Rush, 2000). The CGI-S asks the clinician “Considering your total clinical experience with this particular population, how mentally ill is the patient at this time?” (Busner and Targum, 2007). The CGI-I asks the clinician to assess how much the patient’s illness has improved or worsened relative to the baseline state at the beginning of treatment. Both scales use a seven-point Likert scale (Busner and Targum, 2007). The CGI-S ranges from 1 for “normal, not at all ill” to 7, “among the most extremely ill patients” (Rush, 2000). The CGI-I ranges from 1, “very much improved since the initiation of treatment” to 7, “very much worse since the initiation of treatment” (Rush, 2000).

### 2.1 Data inclusion

All PCMHI patient encounters that occurred from 7 June 2020 through 31 July 2024 were exported from the electronic health record (EHR) to a data file. To account for patients who returned shortly after discharge for the same issue, we defined a treatment cycle as an initial completed visit and at least one completed follow-up visit with at least 90 days between the last follow-up appointment and the next initial appointment. This definition was determined by clinical leaders to be overly conservative and to capture the totality of a patient’s treatment. All patient-specific data from encounters that fell within the date range between the patient’s first and last visits was analyzed as a part of a treatment cycle. Standard protocols were used for patients with no-shows or cancellations. These protocols included discharging patients from care if office staff were unsuccessful in contacting the parent or patient after three attempts.

Patients were included if they had at least one complete treatment cycle. For patients with more than one treatment cycle, the first cycle was used. Diagnoses received throughout treatment were categorized according to the *Diagnostic and Statistical Manual of Mental Disorders*, Fifth Edition (DSM-5) (American Psychiatric Association, 2022) to establish diagnostic groups. Analyses were restricted to patients who received a diagnosis relevant to the conditions screened for by the individual assessments. The majority of patients had multiple overlapping diagnoses, and these groups were not always mutually exclusive.

Variables abstracted from the EHR included all categorical information (age, gender, race, ethnicity, etc) and assessment scores (PHQ-9, GAD-7, PSC-17, or CGI-I) obtained at each visit. Data were sequentially grouped by patient treatment cycle. Assessment scores were taken from the first and last available scores within each cycle. If the patient endorsed the PHQ-9 item assessing suicidal ideation, an alert was generated, and clinical staff contacted the patient immediately to assess safety. The CGI-I scores, provided by clinicians after treatment, evaluated patient illness severity and treatment progress, whereas the PHQ-9, GAD-7, and PSC-17 were self-reported assessments completed by patient (PHQ-9 and GAD-7) or parent/guardians (PSC-17) before the appointment. For patients who did not complete the assessments using their pre-appointment forms, a tablet was provided in the clinic for completion. Not all measures were completed at every visit, and missing assessments were not imputed. Sample sizes reported for each measure reflect only visits at which the respective assessment was completed.

### 2.2 Analysis

The PHQ-9 and GAD-7 were analyzed using both severity score classification and dichotomous cutoffs of >10 (Kroenke et al., 2001; Spitzer et al., 2006; Gilbody et al., 2007; Kroenke et al., 2010). This allowed us to evaluate population-level improvement among our patients using a single threshold, while severity scoring demonstrated graded changes. The PSC-17 subscales used only dichotomous scoring categories, which were used for analysis (Gardner et al., 2007). All analyses were restricted to diagnoses relevant to each measure. Dichotomous analyses were conducted using McNemar’s tests with continuity corrections to assess differences in the proportions of elevated assessment scores before and after treatment. Wilcoxon matched-pairs signed-rank tests were used to evaluate changes within individual elevated-severity scoring groups for the PHQ-9 and GAD-7. Effect sizes are reported as the matched-pairs correlation *r* = *Z*/√*N*, for Wilcoxon tests, where *N* is the number of matched pairs, and as exact odds ratios with 95% confidence intervals for McNemar’s tests. Spearman rank-order correlations between the last CGI-I score and changes in PHQ-9, GAD-7, and PSC-17 subscale scores were calculated. Only the CGI-I was used because the CGI-S is a descriptive characterization of baseline illness severity. Statistical analyses were conducted using Stata 16.1 (College Station, TX).

## 3 Results

The process for selecting patients eligible for analysis is detailed in Figure 1. Of the 4,717 patients initially identified from the EHR, 922 (19.5%) were excluded because they did not meet the definition of a treatment cycle, 896 (19.0%) because they did not have two assessment scores for any single measure, and 197 (4.2%) because they did not have a diagnosis relevant to the measures collected. This resulted in 2,702 patients eligible for analysis (Table 1). A total of 40.2% had a depressive symptom-related diagnosis (*n* = 1,085), 42.7% had an anxiety-related diagnosis (*n* = 1,154), and 48.8% of younger children had symptoms measured by the PSC-17 (*n* = 1,319). These groups constituted the analytic sample for each respective measure. Patients were primarily female, White, Hispanic, and publicly insured. The median patient age was 12 years (interquartile range [IQR] = 8–15 years), and the median number of follow-up appointments was 5 (Bickman et al., 2011; Schweitzer et al., 2023), within the program limits of 6 visits in primary care and 12 visits in the hub setting. The most common diagnosis categories treated in our program were anxiety (30.5%), depression (19.6%), attention-deficit/hyperactivity disorder (ADHD) (13.9%), and adjustment disorders (10.3%). The median CGI-S score at the first visit was 4 (IQR = 3–4), indicating moderate illness. The median CGI-I score at last visit was 3 (IQR = 2–3), indicating minimal improvement. Median assessment scores and changes in scores are presented in Table 2, which demonstrates significant decreases in symptom severity for the PHQ-9, GAD-7, and attention and internalizing PSC-17 subscales.

**Figure 1:**
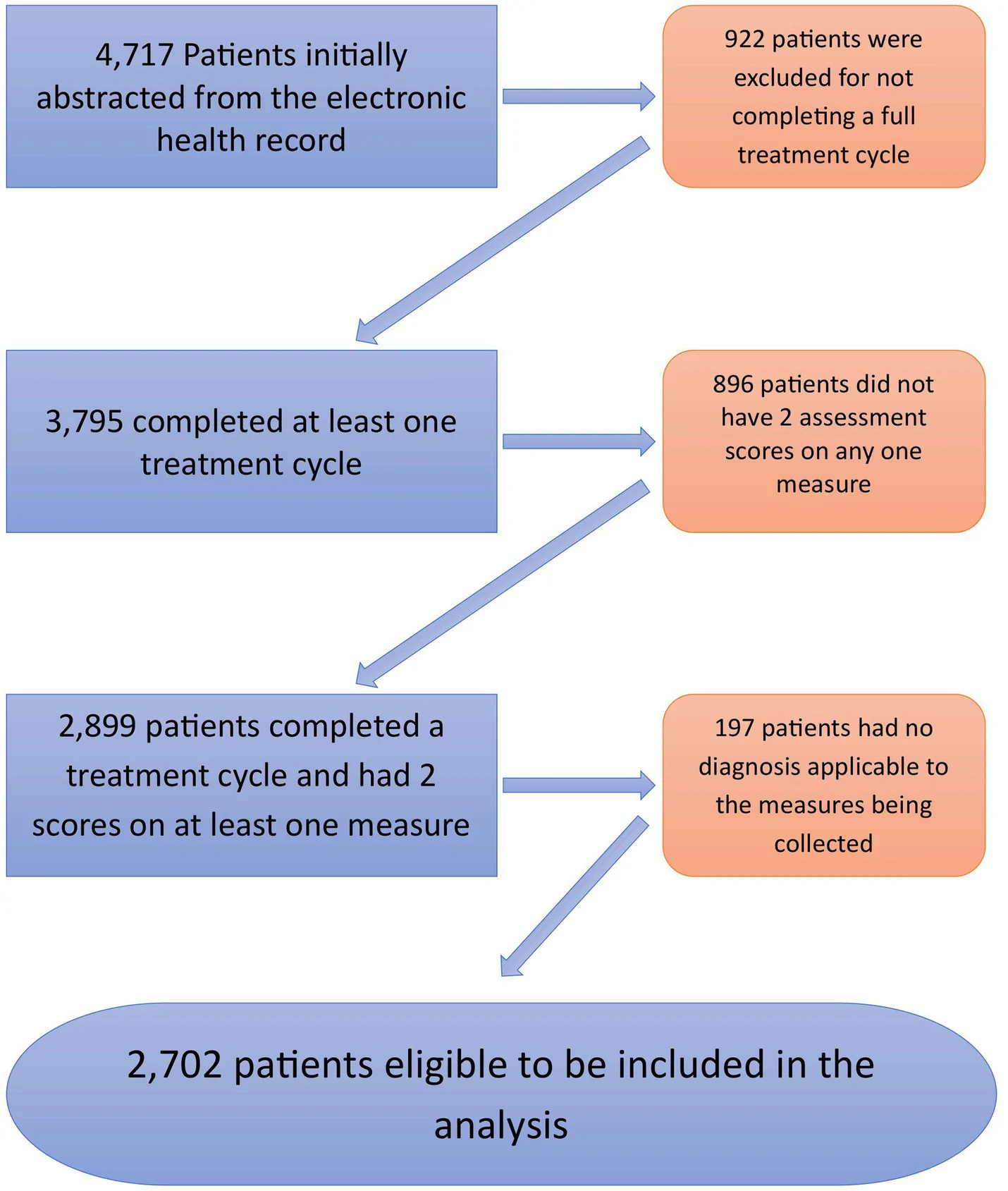
Inclusion criteria process for determining all analysis eligible patients from all patients who had been referred for services in the PCMHI program.

**Table 1:** Demographics characteristics of 2,702 patients treated in a pediatric primary care mental health integration program

Demographics	Data value	
	No.	%
Total	2,702	100.0%
Gender		
Female	1,566	58.0%
Male	1,135	42.0%
Unknown	1	
Race		
White	1,827	68.3%
Hispanic/Latino/Latinx	485	18.1%
Black or African American	142	5.3%
Asian	108	4.0%
Other	76	2.8%
Native Hawaiian or Other Pacific Islander	22	0.82%
American Indian or Alaska Native	15	0.56%
Decline/Unknown	27	
Ethnicity		
Hispanic or Latino	1,377	51.3%
Non-Hispanic	1,309	48.7%
Declined/Unknown	16	
Insurer type		
Public	1,349	60.5%
Private	881	39.5%
Missing/Unknown	472	.
Number of follow-up appointments, Mdn (IQR)	5	(3-9)
Age, Mdn (IQR)	12	(8-15)
Number of measurements during treatment, Mdn (IQR)		
Number of PHQ-9 (*n*=1,085)	3	(3-9)
Number of GAD-7 (*n*=1,154)	6	(3-9)
Number of PSC-17 (*n*=1,319)	4	(3-7)

**Table 2:** Median (IQR) baseline score and change in score and severity score after treatment session.

First measurement severity scoring	First score		Change in score		Change in scoring severity		Effect size (*r*)
PHQ-9							
Minimal (*n*=209)	2	(1, 3)	0	(−2, 3)	0	(0, 1)	NS
Mild (*n*=297)	7	(6, 8)	−2	(−5, 1)	0	(−1, 0)	NS
Moderate (*n*=291)	12	(11, 13)	−4	(−8, −1)	−1	(−2, 0)	0.68
Moderately severe (*n*=186)	16	(15, 18)	−7	(−11, −3)	−2	(−2, −1)	0.77
Severe (*n*=102)	22	(21, 24)	−7.5	(−14, −3)	−2	(−3, 0)	0.80
GAD-7							
Minimal (*n*=242)	2	(1, 4)	0	(−2, 1)	0	(0, 0)	NS
Mild (*n*=412)	7	(6, 8)	−2	(−4, 1)	0	(−1, 0)	NS
Moderate (*n*=302)	12	(11, 13)	−4	(−8, −2)	−1	(−2, 0)	0.70
Severe (*n*=198)	17	(16, 19)	−7	(−12, −2)	−1	(−2, 0)	0.77
PSC-17							
Attention							
Below cutoff (*n*=774)	4	(2, 5)	0	(−1, 1)	0	(0, 0)	
Above cutoff (*n*=545)	8	(7, 9)	−1	(−3, 0)	0	(−1, 0)	
Internalizing							
Below cutoff (*n*=762)	3	(1, 3)	0	(−1, 1)	0	(0, 0)	
Above cutoff (*n*=557)	6	(5, 8)	−1	(−3, 0)	0	(−1, 0)	
Externalizing							
Below cutoff (*n*=940)	3	(1, 5)	0	(−1, 1)	0	(0, 0)	
Above cutoff (*n*=379)	9	(8, 11)	−2	(−4, 0)	0	(−1, 0)	

A negative change indicates that a patient’s total score decreased, and a change in scoring severity category refers to movement between severity categories, with negative values indicating improvement. Sample size: PHQ-9=1,085; GAD-7=1,154; PSC-17=1,319. PHQ-9 and GAD-7 were analyzed using Wilcoxon matched-pairs signed-rank tests. PSC-17 was analyzed using McNemar’s tests with continuity corrections. Effect sizes are reported in Table 3. Effect size(r) was calculated as Z/√N, where N is the number of matched pairs. The reported effect sizes were statistically significant at *p* < 0.001. NS indicates that the severity change was tested but was not statistically significant.

### 3.1 Dichotomous scoring analysis

The proportion of elevated assessment scores decreased significantly after treatment for the PHQ-9 (28.8%; 95% CI: 26.1–31.5%; odds ratio = 4.11, 95% CI, 3.19–5.35), GAD-7 (25.8%; 95% CI, 23.4–28.4%; OR = 4.38, 95% CI, 3.36–5.79), PSC-17 attention subscale (16.5%; 95% CI, 14.6–18.6%; OR = 3.07, 95% CI, 2.34–4.07), and PSC-17 internalizing subscale (17.3%; 15.3–19.4%; OR 1.81, 1.45–2.27) (Table 3). Although the PSC-17 externalizing subscale also decreased (11.0%; 95% CI, 9.4–12.8%; OR = 1.18; 95% CI, 0.92–1.51), the difference in patient-level changes was not statistically significant (Table 3).

**Table 3:** Proportion of screeners above dichotomous scoring cutoffs commonly used by providers at first and last screening with relevant diagnoses

Screeners	% (95% CI)			Odds ratio (95% CI)	*p-*value
	Above cutoff at first score	Moved below cutoff at post	Moved above cutoff at post		
PHQ-9 (*n*=1,085)	53.4 (50.4–56.3)	28.8 (26.1–31.5)	7.0 (5.6–8.7)	4.11 (3.19–5.35)	*p<0.001*
GAD-7 (*n*=1,154)	43.3 (40.5–46.2)	25.8 (23.4–28.4)	5.9 (4.7–7.4)	4.38 (3.36–5.79)	*p<0.001*
PSC-17 (*n*=1,319)					
Attention	41.3 (38.6–44.0)	16.5 (14.6–18.6)	5.4 (4.3–6.7)	3.07 (2.34–4.07)	*p<0.001*
Internalizing	42.2 (39.6–45.0)	17.3 (15.3–19.4)	9.6 (8.1–11.3)	1.81 (1.45–2.27)	*p<0.001*
Externalizing	28.7 (26.4–31.2)	11.0 (9.4–12.8)	9.3 (7.9–11.0)	1.18 (0.92–1.51)	*p=0.19*

Odds ratio compares the odds of moving from above to below the cutoff. Exact confidence intervals. Scoring cutoffs: PHQ-9 and GAD-7 > 10, Attention and Externalizing > 7, Internalizing > 5. Analyzed using McNemar's tests with continuity corrections. CI: confidence interval.

Among the 290 patients who scored >10 on both the GAD-7 and PHQ-9, 35.9% (*n* = 104) had scores that decreased to below 10 on both measures at the end of treatment. An additional 25.5% (*n* = 74) of patients fell below 10 on one measure but not the other, whereas 38.6% of patients (*n* = 112) remained elevated on both measures.

### 3.2 PHQ-9 and GAD-7 scoring severity analysis

Changes in the final PHQ-9 severity score according to the initial severity category are presented in Figure 2. At baseline, 26.8% (*n* = 291) of patients’ PHQ-9 scores were moderate. After treatment, 77.7% (*n* = 226) of these patients had decreased final scores, 6.9% (*n* = 20) had no change in scores, and 15.4% (*n* = 45) had increased scores at the final assessment. These findings demonstrated a significant decrease in the median score after treatment (*z* = 11.58, *p* < 0.001; Table 2). At baseline, 17.2% (*n* = 186) of patient had PHQ-9 scores in the moderately severe category. After treatment, 82.8% (*n* = 154) had decreased scores, 4.3% (*n* = 8) had no change, and 12.9% (*n* = 24) had increased scores. The median score also decreased significantly after treatment (*z* = 10.51, *p* < 0.001; Table 2). Finally, 9.4% (*n* = 102) of patients had PHQ-9 scores in the severe category at baseline. Of these patients, 86.3% (*n* = 88) had decreased final scores, 3.9% (*n* = 4) had no change, and 9.8% (*n* = 10) had increased scores, indicating a significant decrease in scores after treatment (*z* = 8.07, *p* < 0.001; Table 2).

**Figure 2:**
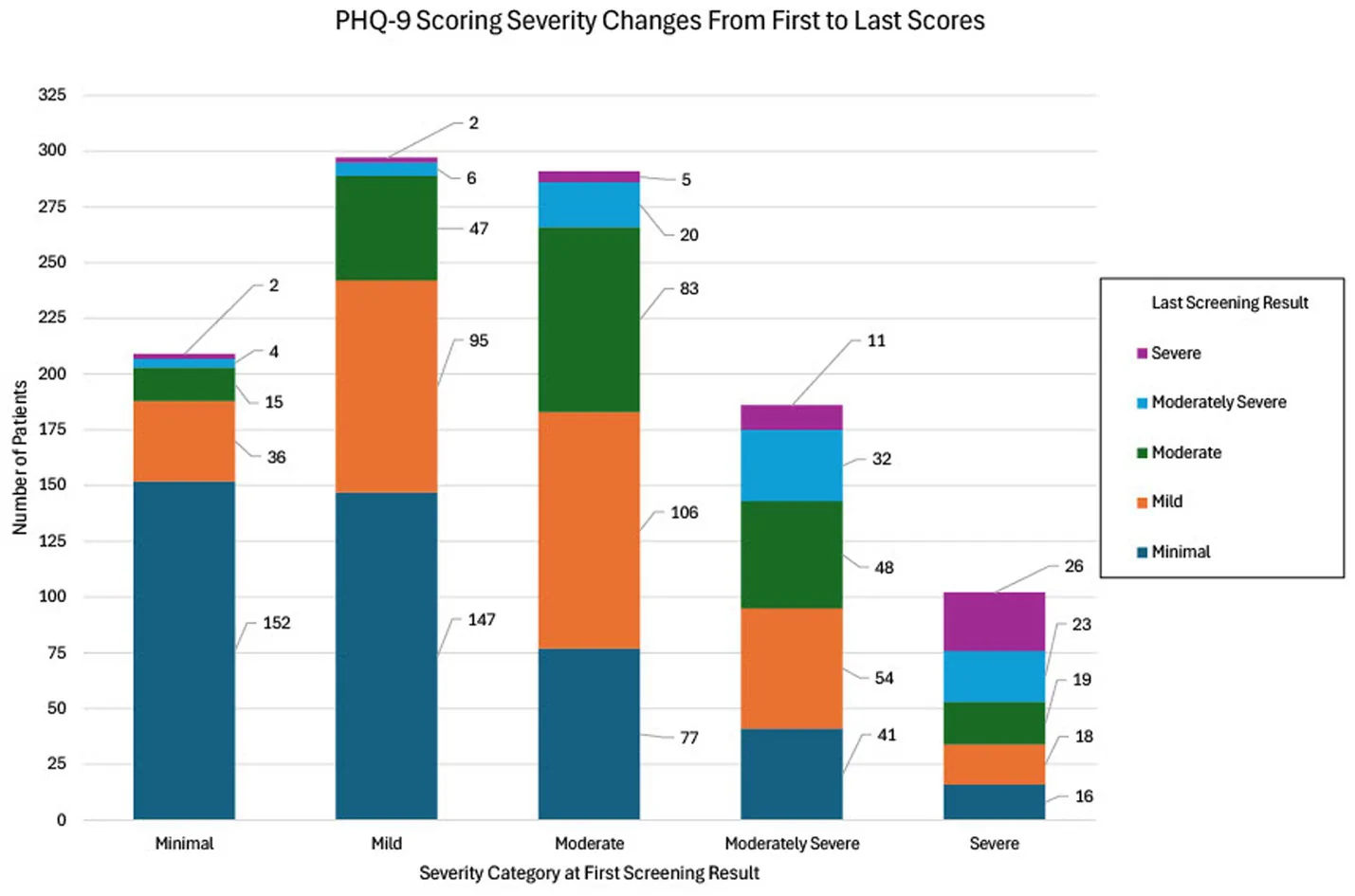
Final PHQ-9 scoring severity by baseline PHQ-9 scoring severity for patients treated in Rady Children’s Transforming mental health integration pediatric primary care clinics, San Diego, CA, June 7, 2020-July 31, 2024.

Changes in final GAD-7 severity scores by initial severity category are presented in Figure 3. At baseline, 26.2% (*n* = 302) of patients had GAD-7 scores in the moderate category. After treatment, 79.8% (*n* = 241) had decreased final scores, 7.0% (*n* = 21) had no change, and 13.2% (*n* = 40) had an increased score category. These findings demonstrated a significant decrease in scores (*z* = 12.11, *p* < 0.001; Table 2). At baseline, 17.2% (*n* = 198) of patients had GAD-7 scores in the severe category. After treatment, 83.3% (*n* = 165) had a decrease in score category, 4.1% (*n* = 8) had no change, and 12.6% (*n* = 25) had increased scores. These findings again demonstrated a significant decrease in scores (*z* = 10.86, *p* < 0.001; Table 2). Effect sizes for the PHQ-9 and GAD-7 are reported in Table 2.

**Figure 3:**
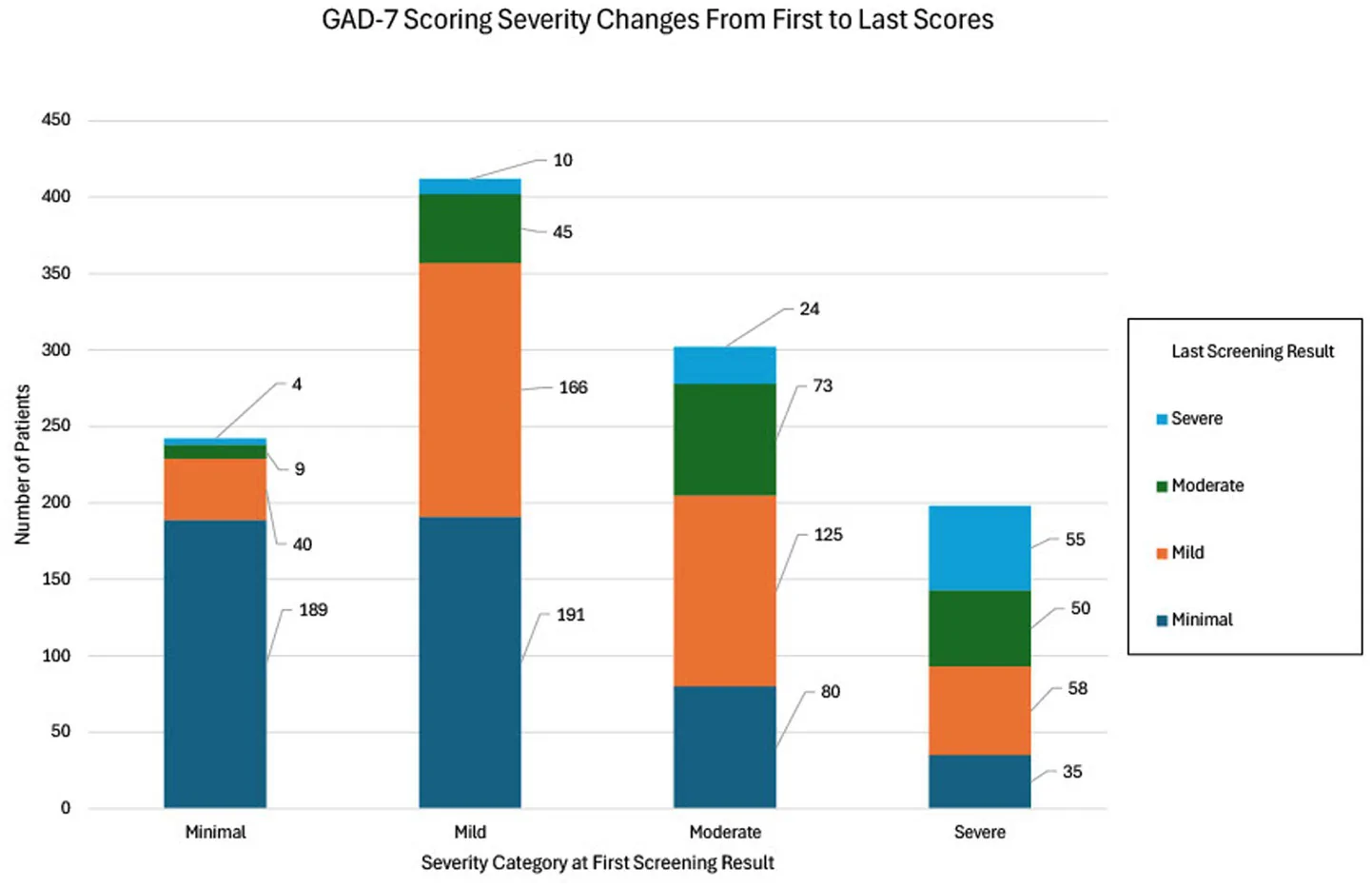
Final GAD-7 scoring severity by baseline GAD-7 scoring severity for patients treated in Rady Children’s primary care mental health integration program primary care clinics, San Diego, CA, June 7, 2020-July 31, 2024.

### 3.3 CGI

There were statistically significant, weak positive correlations between the clinician-rated impression of improvement and changes in patient PHQ-9 and GAD-7 scores, *r_s_*(984) = 0.283 (95% CI, 0.225–0.340; *p* < 0.001) and *r_s_*(1,092) = 0.207 (95% CI, 0.150–0.263; *p* < 0.001), respectively. For the PSC-17 subscales, we detected a weak positive correlation between the therapists’ impression of improvement and changes in patients’ screener scores for attention, internalizing, and externalizing subscales, *r_s_*(1,214) = 0.138 (95% CI, 0.083–0.193; *p* < 0.001), *r_s_*(1,214) = 0.185 (95% CI, 0.130–0.239; *p* < 0.001), and *r_s_*(1,214) = 0.102 (95% CI, 0.046–0.157; *p* < 0.001), respectively.

## 4 Discussion

PCMHI primarily serves children and adolescents experiencing mild to moderate symptoms of anxiety and depression or behavioral challenges. An analysis of the measures used by our program to monitor patients and supplement clinical information relevant to these presenting problems revealed that symptom scores decreased for many patients over the course of treatment.

Data collection began in June 2020, during the COVID-19 pandemic, which may have affected both baseline severity and symptom trajectory independent of treatment. Because the cohort spans the acute pandemic period and its aftermath, changes in symptom burden and service delivery represent potential sources of variation that we could not separate. The correlations we observed between CGI-I scores and other measures were similar to those reported by other studies. The CGI-I is a useful clinician-reported measure for tracking broad changes across various patient populations (Jiang and Ahmed, 2009; Masand et al., 2011; Zhang et al., 2022).

It has been reported that 93% of patients with PHQ-9 scores <10 do not have a depressive disorder (Kroenke et al., 2001). It is encouraging that 28.8% of all patients analyzed had scores that decreased below the cutoff of 10 after short-term treatment. For the GAD-7, 89% of patients with scores >10 are characterized as having generalized anxiety disorders (Spitzer et al., 2006). It was also encouraging that, among all patients analyzed, 25.8% had scores that decreased below the cutoff at their final assessment.

Other studies have attempted to define clinical success when evaluating reductions in assessment scores. A 50% reduction in PHQ-9 scores has been proposed as a target for successful depression treatment because it indicates clinical improvement, is easy to calculate, and does not favor higher or lower baseline symptom severity (Coley et al., 2020). We did not adopt percent reduction as an outcome metric because movement across validated severity categories and the clinical cutoff more directly reflects the thresholds used by our clinicians to guide care. For the GAD-7, a reduction of 6 points and crossing a severity cutoff have been proposed as indicators of meaningful change (Bischoff et al., 2020). We achieved these criteria in our patients with anxiety diagnoses whose baseline scores place them in the severe scoring severity group. Median score reductions were larger among patients with higher baseline severity, ranging from 4 points in the moderate group to 7.5 in the severe group for the PHQ-9 (Table 2). Regression to the mean and restricted range of change among patients with lower baseline scores are plausible contributors to this pattern, which we did not test directly.

For the PSC-17, we identified statistically significant reductions for two subscales, but the magnitude of these changes were smaller than the changes we found for the PHQ-9 or GAD-7 (Table 3). The externalizing subscale showed no statistically significant changes. Treatment, and patient-related factors that may impact this domain merit further investigation. The proportion of patients who scored above the externalizing cutoff at their first score among our patients was substantially lower when compared to other measures for our patients (Table 3). Changes on the subscale of two or more points and crossing the dichotomous cutoff has been reported to reflect reliable change (Murphy et al., 2016; Kamin et al., 2015). We did identify significant statistical changes on two of our subscales, but did not achieve the level of change suggested by others.

Within our program, MIC assessments demonstrated the usefulness of monitoring changes in symptoms throughout treatment. The PHQ-9, GAD-7, and PSC-17 were completed prior to appointments and provided updated information regarding patient status without using limited time with the clinician. Members of our clinical team routinely use these measures to track patient progress. The CGI differs from the PHQ-9, GAD-7 and PSC-17 as it is completed by the clinician rather than the patient or caregiver. These tools each provide valuable information, particularly for patients whose conditions lack diagnostic clarity. It was encouraging to validate that the patient-reported assessments were significantly, albeit weakly, correlated with the clinician CGI findings.

This study did have limitations. The results observed cannot be determined to be causal; however, the decrease in anxiety and depression symptoms associated with timing of patient treatment is encouraging. Data points utilized for these analyses were the first and last measures recorded within the treatment cycle for each patient. It is possible that scores may have varied more greatly during treatment than recorded at first and last appointments. While there was a limit of 6 follow-up visits in the primary care setting and 12 in the hub setting, some patients received additional treatment due to rapid re-referrals and clinicians requesting treatment extensions. This potentially may have introduced some variability into the treatment length among patients.

A further limitation is the availability of validated brief assessments across behavioral health conditions. As a result, our analysis was limited to the conditions for which the PHQ-9, GAD-7 and PSC-17 are applicable. These conditions do make up the majority of issues adolescents are referred for. Further investigation into potential differences that the number of visits might have on patient scoring outcomes may be warranted. All patients were treated within a single pediatric health network, and analyses were restricted to patients with at least two scores on a given measure. The results may therefore not apply to programs structured differently or to patients who disengaged earlier. Finally, it is possible that CGI-I scores entered by the clinicians may have been biased by observed changes on the other measures.

Future areas of investigation include analyzing score changes with respect to other potentially confounding variables (e.g., psychiatric medication, school related treatment, and/or access to school-based mental health support) and exploring implementation strategies to enhance clinical team use of data to monitor and modify treatment. Our study demonstrated self- and clinician-reported changes in symptoms over brief treatment periods. We have added the benefit of electronic assessments being captured in the EMR. For patients who have not completed the assessment before the appointment, clinicians often ask patients to complete on a tablet at the start of the appointment. We do not know the proportion of patients who completed assessments before the appointment or at the start of the appointment.

MIC assessments can be effectively used in a PCBH-based integrated care model for children and adolescents. Our patients completed these measures prior to appointments, minimally impacting face-to-face therapeutic time. In addition, treating clinicians observed clinical improvements in agreement with the reduction in scores indicated by the CGI-I positive correlations. Although these correlations were weak, they demonstrated their value as a single data point for patient monitoring and displayed the potential benefit of incorporating clinician rating instruments into standardized MIC. Symptom improvement and clinician observation reinforce the PCMHI model and provide opportunities for clinicians to use MIC data in their case formulation and clinical decision-making. These tools also provide supervisors with discrete clinical data when reviewing patient cases with care team members.

## Data Availability

The data that support the findings of this study are available on a reasonable request from the corresponding author, KH. The data are not publicly available due to privacy and ethical restrictions.

## References

[ref1] American Psychiatric Association (2022). Diagnostic and Statistical Manual of Mental Disorders. DSM-5-TR. Washington, DC: American Psychiatric Association Publishing.

[ref2] Asarnow J. R. Rozenman M. Wiblin J. Zeltzer L. (2015). Integrated medical-behavioral care compared with usual primary care for child and adolescent behavioral health: a meta-analysis. JAMA Pediatr. 169:929. doi: 10.1001/jamapediatrics.2015.1141, 26259143

[ref3] Bickman L. Kelley S. D. Breda C. De Andrade A. R. Riemer M. (2011). Effects of routine feedback to clinicians on mental health outcomes of youths: results of a randomized trial. PS 62, 1423–1429. doi: 10.1176/appi.ps.002052011, 22193788

[ref4] Bischoff T. Anderson S. R. Heafner J. Tambling R. (2020). Establishment of a reliable change index for the GAD-7. Psychol. Community Health 8, 176–187. doi: 10.5964/pch.v8i1.309

[ref5] Boswell J. F. Hepner K. A. Lysell K. Rothrock N. E. Bott N. Childs A. W. . (2023). The need for a measurement-based care professional practice guideline. Psychotherapy 60, 1–16. doi: 10.1037/pst0000439, 35771518

[ref6] Busner J. Targum S. D. (2007). The clinical global impressions scale: applying a research tool in clinical practice. Psychiatry (Edgmont) 4, 28–37. PMC2880930 20526405

[ref7] Coley R. Y. Boggs J. M. Beck A. Hartzler A. L. Simon G. E. (2020). Defining success in measurement-based Care for Depression: a comparison of common metrics. PS 71, 312–318. doi: 10.1176/appi.ps.201900295, 31847739

[ref8] Gardner W. Lucas A. Kolko D. J. Campo J. V. (2007). Comparison of the PSC-17 and alternative mental health screens in an at-risk primary care sample. J. Am. Acad. Child Adolesc. Psychiatry 46, 611–618. doi: 10.1097/chi.0b013e318032384b, 17450052

[ref9] Gilbody S. Richards D. Barkham M. (2007). Diagnosing depression in primary care using self-completed instruments: UK validation of PHQ-9 and CORE-OM. Br. J. Gen. Pract. 57, 650–652. PMC2099671 17688760

[ref10] Jellinek M. S. Murphy J. M. Burns B. J. (1986). Brief psychosocial screening in outpatient pediatric practice. J. Pediatr. 109, 371–378. doi: 10.1016/S0022-3476(86)80408-5, 3734977

[ref11] Jellinek M. S. Murphy J. M. Little M. Pagano M. E. Comer D. M. Kelleher K. J. (1999). Use of the pediatric symptom checklist to screen for psychosocial problems in pediatric primary care: a national feasibility study. Arch. Pediatr. Adolesc. Med. 153, 254–260. doi: 10.1001/archpedi.153.3.254, 10086402 PMC3905751

[ref12] Jensen-Doss A. Douglas S. Phillips D. A. Gencdur O. Zalman A. Gomez N. E. (2020). Measurement-based care as a practice improvement tool: clinical and organizational applications in youth mental health. Evid.-Based Pract. Child Adolesc. Mental Health 5, 233–250. doi: 10.1080/23794925.2020.1784062, 33732875 PMC7962174

[ref13] Jensen-Doss A. Weisz J. R. (2008). Diagnostic agreement predicts treatment process and outcomes in youth mental health clinics. J. Consult. Clin. Psychol. 76, 711–722. doi: 10.1037/0022-006X.76.5.711, 18837589

[ref14] Jiang Q. Ahmed S. (2009). An analysis of correlations among four outcome scales employed in clinical trials of patients with major depressive disorder. Ann. General Psychiatry 8:4. doi: 10.1186/1744-859X-8-4, 19166588 PMC2645397

[ref15] Kamin H. S. McCarthy A. E. Abel M. R. Jellinek M. S. Baer L. Murphy J. M. (2015). Using a brief parent-report measure to track outcomes for children and teens with internalizing disorders. Child Psychiatry Hum. Dev. 46, 851–862. doi: 10.1007/s10578-014-0525-8, 25476666

[ref16] Klein J. B. Lavigne J. V. Seshadri R. (2010). Clinician-assigned and parent-report questionnaire-derived child psychiatric diagnoses: correlates and consequences of disagreement. Am. J. Orthopsychiatry 80, 375–385. doi: 10.1111/j.1939-0025.2010.01041.x, 20636943

[ref17] Kroenke K. Spitzer R. L. Williams J. B. W. (2001). The PHQ-9: validity of a brief depression severity measure. J. Gen. Intern. Med. 16, 606–613. doi: 10.1046/j.1525-1497.2001.016009606.x, 11556941 PMC1495268

[ref18] Kroenke K. Spitzer R. L. Williams J. B. W. Löwe B. (2010). The patient health questionnaire somatic, anxiety, and depressive symptom scales: a systematic review. Gen. Hosp. Psychiatry 32, 345–359. doi: 10.1016/j.genhosppsych.2010.03.006, 20633738

[ref19] Lewis C. C. Boyd M. Puspitasari A. Navarro E. Howard J. Kassab H. . (2019). Implementing measurement-based Care in Behavioral Health: a review. JAMA Psychiatry 76, 324–335. doi: 10.1001/jamapsychiatry.2018.3329, 30566197 PMC6584602

[ref20] Liu F. F. Adrian M. C. (2019). Is treatment working? Detecting real change in the treatment of child and adolescent depression. J. Am. Acad. Child Adolesc. Psychiatry 58, 1157–1164. doi: 10.1016/j.jaac.2019.02.011, 30825497 PMC11488664

[ref21] Masand P. O’Gorman C. Mandel F. S. (2011). Clinical global impression of improvement (CGI-I) as a valid proxy measure for remission in schizophrenia: analyses of ziprasidone clinical study data. Schizophr. Res. 126, 174–183. doi: 10.1016/j.schres.2010.10.024 21185155

[ref22] Mossman S. A. Luft M. J. Schroeder H. K. Varney S. T. Fleck D. E. Barzman D. H. . (2017). The generalized anxiety disorder 7-item scale in adolescents with generalized anxiety disorder: signal detection and validation. Ann. Clin. Psychiatry 29, 227–234. doi: 10.1177/104012371702900404, 29069107 PMC5765270

[ref23] Murphy J. M. Bergmann P. Chiang C. Sturner R. Howard B. Abel M. R. . (2016). The PSC-17: subscale scores, reliability, and factor structure in a new national sample. Pediatrics 138:e20160038. doi: 10.1542/peds.2016-0038, 27519444 PMC5005018

[ref24] Reiter J. T. Dobmeyer A. C. Hunter C. L. (2018). The primary care behavioral health (PCBH) model: an overview and operational definition. J. Clin. Psychol. Med. Settings 25, 109–126. doi: 10.1007/s10880-017-9531-x, 29480434

[ref25] Rush A. J. (2000). Handbook of Psychiatric Measures. Washington, DC: American Psychiatric Association, 820.

[ref26] Schweitzer J. Bird A. Bowers H. Carr-Lee N. Gibney J. Schellinger K. . (2023). Developing an innovative pediatric integrated mental health care program: interdisciplinary team successes and challenges. Front. Psych. 14:1252037. doi: 10.3389/fpsyt.2023.1252037, 38045623 PMC10693412

[ref27] Scott K. Lewis C. C. (2015). Using measurement-based care to enhance any treatment. Cogn. Behav. Pract. 22, 49–59. doi: 10.1016/j.cbpra.2014.01.010, 27330267 PMC4910387

[ref28] Spitzer R. L. Kroenke K. Williams J. B. W. Löwe B. (2006). A brief measure for assessing generalized anxiety disorder: the GAD-7. Arch. Intern. Med. 166:1092. doi: 10.1001/archinte.166.10.1092 16717171

[ref29] Zhang C. Y. Voort J. L. V. Yuruk D. Mills J. A. Emslie G. J. Kennard B. D. . (2022). A characterization of the clinical global impression scale thresholds in the treatment of adolescent depression across multiple rating scales. J. Child Adolesc. Psychopharmacol. 32, 278–287. doi: 10.1089/cap.2021.0111, 35704877 PMC9353998

